# Transcatheter Aortic Valve Implantation in Low-Risk and Younger Patients with Porcelain Aorta: A State-of-the-Art Narrative Review

**DOI:** 10.3390/medicina62030483

**Published:** 2026-03-04

**Authors:** Nikoleta Stanitsa, Michalis Tsibinos, Emmanouel Tempelis, Orestis Paliaroutas, Grigoris Trikas, Ilias Samiotis, Panagiotis Dedeilias

**Affiliations:** 1 Department of Cardiac Surgery, Evangelismos General Hospital, 10676 Athens, Greecedremmanouiltempe@outlook.com (E.T.); samiotisilias@gmail.com (I.S.); pdedeilias@gmail.com (P.D.); 2 1st Orthopedic Surgery Department, KAT General Hospital, 14561 Athens, Greece; orestis.paliaroutas21@gmail.com; 3 Department of Cardiology, Nimitz Hospital, 11521 Athens, Greece; gr-trikas@hotmail.com

**Keywords:** porcelain aorta, transcatheter aortic valve implantation, aortic stenosis, low surgical risk, stroke, cerebral embolic protection, structural valve deterioration, lifetime management

## Abstract

*Background and Objectives:* Porcelain aorta is an anatomy-driven high-risk phenotype characterized by extensive calcification of the ascending aorta, which complicates surgical aortic valve replacement by increasing embolic and technical hazards during cannulation and cross-clamping. As transcatheter aortic valve implantation (TAVI) expands into younger and low-surgical-risk populations, porcelain aorta creates a distinct clinical dilemma: optimizing short-term procedural safety while ensuring durable long-term outcomes and preserving future treatment options. *Materials and Methods*: We performed a targeted literature search of MEDLINE/PubMed, EMBASE, and the Cochrane Central Register of Controlled Trials (CENTRAL), with the last search conducted on 31 January 2026. We synthesized contemporary clinical evidence on TAVI in patients with imaging-defined porcelain aorta, focusing on neurological outcomes, procedural strategies to reduce embolic risk, access considerations, valve performance, cerebral embolic protection, and implications for lifetime valve management (including coronary access and feasibility of future valve-in-valve interventions). *Results*: The evidence base specific to porcelain aorta in the contemporary TAVI era is limited and largely observational. Across published cohorts, TAVI avoids direct ascending aortic cannulation and cross-clamping and is generally associated with favorable early safety, with a recurring directional signal toward lower neurological risk compared with surgical strategies that require manipulation of a severely calcified ascending aorta. Interpretation is constrained by heterogeneity in porcelain-aorta definitions, patient selection, valve platforms and access routes, as well as, variability in neurological endpoint definitions and adjudication. *Conclusions*: In patients with porcelain aorta, TAVI is frequently favored because it minimizes ascending aortic manipulation and may mitigate neurological and procedural hazards. In younger and low-risk patients, Heart Team decision-making should incorporate lifetime management principles, including access planning, preservation of future coronary access, and procedural strategies to reduce embolic risk (with consideration of cerebral embolic protection when appropriate).

## 1. Introduction

Porcelain aorta (PA) is an extreme manifestation of thoracic aortic atherosclerosis, characterized by dense calcification of the ascending aorta that frequently extends into the aortic arch [1]. Although PA is classically associated with advanced age and diffuse atherosclerotic burden, it is increasingly detected during contemporary valve work-up on pre-procedural computed tomography (CT), including in patients who otherwise appear low-risk by conventional surgical scores [2]. In such cases, anatomy-driven hazards may dominate peri-procedural risk, particularly with respect to embolic neurologic events and the technical feasibility of surgical manipulation (e.g., cannulation and cross-clamping) [3]. Standard risk models (e.g., STS-PROM, EuroSCORE II) primarily reflect comorbidity burden and physiological reserve and may therefore underestimate anatomy-driven impediments such as PA, complex arch atheroma, or hostile access routes [4]. Beyond traditional comorbidities, earlier and more aggressive calcific atherosclerosis phenotypes may also be influenced by non-traditional modifiers (e.g., chronic stress exposure and adverse social determinants affecting diet), which may help contextualize why hostile aortic anatomy is sometimes encountered even in apparently low-risk patients [5].

**Figure 1:**
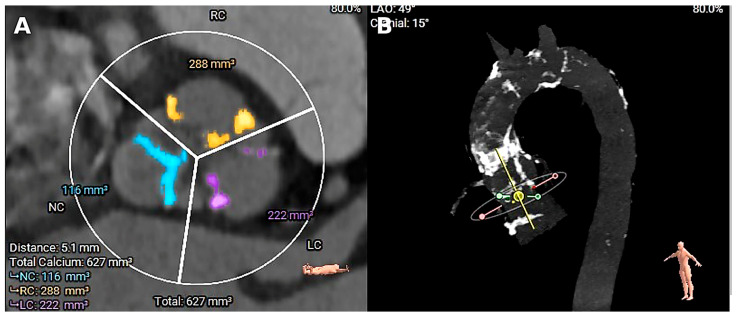
Computed tomography characterization of porcelain aorta and calcific burden relevant to procedural risk. (**A**) Short-axis multislice computed tomography (“hockey-puck”) view of the aortic valve demonstrating asymmetric leaflet calcification with quantified calcium burden relevant to embolic risk and transcatheter valve deployment. (**B**) Three-dimensional volume-rendered computed tomography image showing extensive, circumferential calcification of the ascending aorta and proximal aortic arch, consistent with a porcelain aorta and highlighting the prohibitive risk of surgical aortic manipulation.

Across studies, PA definitions vary (circumferential versus segmental calcification; isolated ascending aortic involvement versus arch extension), and imaging modalities range from CT angiography to fluoroscopy and intraoperative assessment (Figure 1) [6]. Because the extent and location of calcification influence procedural planning (e.g., feasibility of cross-clamping, cannulation strategy, and consideration of embolic protection), we summarize study-level definitions and distribution patterns in Table 1 [7]. For the purposes of this review, we adopt a pragmatic CT-based working definition: “classic” PA refers to near-circumferential calcification of the ascending aorta (typically >270° arc on axial images) over a clinically meaningful segment, with or without arch involvement; segmental or non-circumferential calcification is described separately where reported [8,9]. When studies apply different thresholds, we preserve the authors’ definitions and summarize anatomic extent accordingly in Table 1.

The management of severe symptomatic aortic stenosis (AS) has shifted substantially over the last decade. In the PARTNER 3 trial, transcatheter aortic valve implantation (TAVI) with a balloon-expandable valve was shown to be an effective alternative to surgical aortic valve replacement (SAVR) in low-surgical-risk patients [10]. Similarly, the Evolut Low Risk Trial demonstrated noninferiority of self-expanding TAVI compared with SAVR in low-risk candidates [11]. However, global surgical risk scores are imperfect surrogates of procedural complexity and may fail to capture anatomy-driven barriers that meaningfully increase operative risk. Among these, PA remains one of the most significant and technically challenging anatomic conditions encountered in modern valve practice. In SAVR, routine steps—such as aortic cannulation, cross-clamping, and aortotomy—may be hazardous in the setting of heavy ascending aortic calcification, increasing the risks of embolic stroke, iatrogenic aortic injury, and major bleeding [12]. To mitigate these hazards, surgeons have historically employed alternative strategies (e.g., “no-touch” techniques and deep hypothermic circulatory arrest) aimed at minimizing aortic manipulation; however, these approaches remain invasive and may not fully eliminate embolic risk [12].

A growing dilemma arises when PA is identified in younger patients otherwise classified as low-surgical-risk: anatomical risk may outweigh clinical risk, challenging the traditional preference for SAVR based on durability, while TAVI minimizes aortic manipulation but raises concerns regarding long-term valve durability, future coronary access, and lifetime management [13]. In this context, we provide a narrative review of contemporary evidence regarding TAVI outcomes in low-risk and/or younger patients with CT-confirmed PA, with emphasis on neurological outcomes, valve performance, the role of cerebral embolic protection devices, and lifetime management considerations in this anatomically complex population.

## 2. Methods

This manuscript is a state-of-the-art narrative review synthesizing contemporary evidence on transcatheter aortic valve implantation (TAVI) in patients with porcelain aorta (PA), with emphasis on: (i) younger and/or low-surgical-risk profiles; (ii) neurological outcomes; (iii) procedural strategies to reduce embolic risk; and (iv) implications for lifetime valve management.

We performed a structured literature search of MEDLINE (via PubMed), EMBASE, and the Cochrane Central Register of Controlled Trials (CENTRAL). The last search was completed on 31 January 2026. We focused primarily on the contemporary device era (1 January 2015 onward) to reflect current valve platforms, imaging practice, and procedural techniques. Earlier PA studies were additionally considered when they provided durable, high-yield information on surgical strategies or clinically relevant outcome signals that remain applicable to contemporary decision-making. Full database-specific search strategies and limits are provided in Appendix A.

We included peer-reviewed, English-language, human studies reporting outcomes after TAVI and/or surgical strategies in patients with PA defined by imaging or intraoperative assessment. We prioritized cohorts with clear involvement of the ascending aorta (with or without arch extension), particularly when PA was defined by computed tomography (CT). Studies using fluoroscopy and/or intraoperative findings were eligible when the report explicitly described severe/heavily calcified (“porcelain”) ascending aortic disease affecting feasibility or safety of cannulation and/or cross-clamping. Studies were prioritized when neurological outcomes were reported (clinical stroke and/or surrogate embolic endpoints). We also included studies informing lifetime management (hemodynamic performance, durability signals, coronary access, and feasibility of future interventions), particularly in younger and/or low-risk contexts. Case reports and small case series (<10 patients) were excluded unless they provided unique technical insights not otherwise available (e.g., novel access routes, embolic-risk mitigation strategies, or feasibility considerations specific to PA).

Two reviewers independently screened records and extracted prespecified variables: study design and setting; sample size; patient profile (age and surgical risk, when reported); PA definition and anatomic extent; imaging modality; TAVI access route and valve platform; comparator strategy (standard SAVR, no-touch approaches, deep hypothermic circulatory arrest, and/or hybrid strategies); endpoint definitions; and follow-up duration. Discrepancies were resolved by consensus. When multiple publications appeared to report overlapping cohorts, outcomes were extracted preferentially from the most complete and/or most recent report.

### Quality Appraisal and Synthesis Approach

Study characteristics and quality appraisal are summarized in Table 1, Table 2 and Table 3. Observational cohort studies were appraised using the Newcastle–Ottawa Scale (NOS) to contextualize inference rather than as exclusion criteria; study-level NOS summaries are reported in Table 3. The clinical evidence base specific to PA in the contemporary TAVI era is limited and heterogeneous; key PA-focused cohorts/series informing phenotype-specific and/or comparative outcome assessment are summarized in Table 2 and complemented by mechanistic, access-strategy, embolic-protection, and durability literature for contextual synthesis. Given heterogeneity in PA definitions and anatomic distribution, procedural era and device platforms, comparator surgical strategies, endpoint definitions/adjudication, and potential cohort overlap, we did not perform a quantitative meta-analysis. Outcomes are summarized descriptively using study-level values and ranges, and major sources of clinical and methodological heterogeneity are highlighted.

**Table 1:** Definitions and anatomical extent of porcelain aorta across key studies.

Study	Imaging Modality Used to Define PA	Operational Definition (As Reported)	Anatomical Extent Reported	Notes
Zahn 2013 [14]	Variable by center (registry-based)	Site-/center-reported “porcelain ascending aorta”/severe aortic atherosclerosis; no uniform CT threshold	Ascending aorta; arch involvement not reported	High heterogeneity expected; pragmatic/operator-dependent definition
Ramírez-Del Val 2018 [12]	CT reported	PA identified during operative evaluation; explicit threshold not uniformly specified	Ascending ± arch not clearly specified	Comparative cohort; selection bias likely
Byrne 1998 [15]	Intraoperative assessment	“Porcelain aorta” requiring clampless strategy	Ascending aorta (surgical target of avoidance)	Strategy-driven definition; informs surgical feasibility discussion
Lauten 2025 [16]	CT angiography	CT-confirmed PA (definition per authors)	Ascending aorta; arch involvement not consistently reported	Clinically relevant early stroke signal; supports embolic-risk mitigation consideration

**Abbreviations:** CT, computed tomography; PA, porcelain aorta.

**Table 2:** Key clinical studies informing management of porcelain aorta in TAVI and surgical strategies.

Study	Design/Setting	Population	PA Definition/Imaging	Strategy/Comparator	Neurological Outcomes Reported	Follow-Up
Zahn et al., 2013 [14]	Multicenter registry (“real-world”)	Severe AS undergoing TAVI (PA vs. non-PA subgroup within TAVI cohort)	Registry/site-reported “porcelain ascending aorta”/severe aortic atherosclerosis; no standardized CT threshold	Within TAVI: PA vs. non-PA	Clinical stroke (as reported); early outcomes	30 days
Ramírez-Del Val et al., 2018 [12]	Observational comparative cohort	Severe AS with PA undergoing valve intervention	PA identified during operative evaluation (CT reported; criteria not uniformly specified)	TAVI vs. SAVR	Clinical stroke; early mortality	30 days
Byrne et al., 1998 [15]	Surgical series	Severe AS with porcelain aorta undergoing AVR	Intraoperative recognition of porcelain ascending aorta	AVR using DHCA “no-touch/clampless” technique	Neurologic events (as reported)	In-hospital
Lauten et al., 2025 [16]	Observational cohort	Transfemoral balloon-expandable TAVI (PA vs. non-PA)	CT angiography-confirmed PA (definition per authors)	Within transfemoral TAVI: PA vs. non-PA	Early stroke (≤72 h) and 30-day outcomes (as reported)	30 days

**Abbreviations:** AS, aortic stenosis; AVR, aortic valve replacement; CT, computed tomography; DHCA, deep hypothermic circulatory arrest; PA, porcelain aorta; SAVR, surgical aortic valve replacement; TAVI, transcatheter aortic valve implantation. Note: Where studies did not apply standardized PA thresholds or formal neurologic endpoint adjudication, this is reflected in the definition/design fields.

**Table 3:** Risk-of-bias appraisal (Newcastle–Ottawa Scale summary) for observational cohort studies.

Study	Selection	Comparability	Outcome/Follow-Up	Overall Judgment
Zahn 2013 [14]	Moderate	Low	Moderate	Moderate risk (registry design; residual confounding; non-standard PA definition)
Ramírez-Del Val 2018 [12]	Moderate	Low	Moderate	Moderate–high risk (nonrandomized comparator; confounding by indication)
Lauten 2025 [16]	Moderate	Low	Moderate	Moderate risk (observational; endpoint definitions/timing may differ)
Byrne 1998 [15]	—	—	—	Not scored by NOS (non-comparative surgical series)

**Notes:** NOS ratings were used to contextualize inference and were not applied as exclusion criteria. “—” indicates NOS domains not applicable to a non-comparative surgical case series. PA: Porcelanoid Aorta.

## 3. Results

The clinical evidence on TAVI in patients with porcelain aorta remains largely derived from observational cohorts and registry-based analyses rather than randomized trials, with PA defined variably by CT, fluoroscopy, and/or intraoperative assessment.

In the contemporary device era, the porcelain-aorta-specific outcome literature is relatively small; four key clinical cohorts/series that explicitly evaluated the PA phenotype and/or compared treatment strategies are summarized in Table 2, while additional mechanistic, access, embolic-protection, and durability studies are incorporated to contextualize interpretation.

This is expected, as porcelain aorta represents a high-risk anatomical substrate in which randomization to strategies requiring extensive aortic manipulation may be ethically and practically difficult. Across published reports, the diagnosis is typically established by multislice CT, but the operational definition is not uniform: some studies require circumferential (“ring-like”) calcification of the ascending aorta, while others use broader thresholds that include extensive anterior or patchy calcification. These definitional differences are clinically important because they influence procedural planning, baseline embolic risk, feasibility of surgical clamping/cannulation, and the “true” degree of hostility of the ascending aorta.

Comparative evidence most commonly evaluates TAVI against conventional SAVR or against modified surgical strategies intended to reduce manipulation of the calcified ascending aorta (e.g., no-touch techniques, alternative cannulation sites, or deep hypothermic circulatory arrest) [13,15]. These surgical approaches are not equivalent, and differences in technique selection and baseline anatomy can confound simple ‘TAVI vs. surgery’ comparisons; we therefore describe outcomes with attention to the surgical strategy when such detail is available.

Neurological injury is the outcome most tightly linked to the porcelain-aorta phenotype [16]. The core mechanistic concern is that instrumentation or manipulation in the setting of extensive ascending aortic calcification can mobilize particulate debris and trigger cerebral embolization [17,18]. In comparative observational cohorts of patients with porcelain aorta, neurological event rates after TAVI are generally reported as low, and available studies often suggest a directional signal favoring TAVI over surgical strategies in this anatomically high-risk setting [18,19]. However, interpretation is limited by substantial between-study heterogeneity: neurological endpoints are variably defined (e.g., disabling vs. non-disabling stroke; clinical stroke vs. imaging-detected lesions), adjudication practices are inconsistent, and baseline risk is rarely balanced between treatment groups [20]. Accordingly, the most defensible conclusion is not that TAVI “proves superiority,” but that the accumulated observational evidence is consistent with a biologically plausible principle—namely, that reducing direct ascending aortic manipulation may mitigate embolic hazard in heavily calcified aortas [19,20].

Mechanistic investigations reinforce this concept. Studies using transcranial Doppler and other surrogate markers of cerebral embolization demonstrate that embolic signals can occur during cardiac surgery even when teams attempt to minimize direct aortic handling. This likely reflects the fact that embolic material may still arise from essential steps such as arterial cannulation and cardiopulmonary bypass (CPB), where altered flow patterns, turbulence, and shear forces can mobilize debris or generate emboli within diseased great vessels [19]. Accordingly, neurological risk is not simply a binary function of “clamp versus no clamp,” but rather reflects the cumulative burden of aortic/great-vessel manipulation together with the hemodynamic stresses imposed during CPB [21].

Early mortality and major morbidity in porcelain aorta are strongly influenced by anatomy-driven technical constraints. TAVI, by design, avoids cross-clamping and direct ascending aortic cannulation, which may reduce several procedure-specific hazards that become amplified in a severely calcified aorta. Observational reports frequently describe favorable early outcomes after TAVI in this phenotype, particularly when the procedure can be performed through transfemoral or other less invasive access routes with careful imaging-based planning.

In contrast, surgical strategies for severe aortic stenosis in the setting of porcelain aorta often require advanced technical modifications, and perioperative risk may be driven by challenges that are less prominent in routine SAVR. Surgical reports emphasize bleeding from fragile, calcified tissue, difficulty identifying safe cannulation and cross-clamp sites, and—in selected cases—added risk associated with alternative perfusion strategies or circulatory arrest used to minimize aortic manipulation. These issues do not imply that surgery is “inappropriate,” but they underscore why porcelain aorta is historically treated as a major risk modifier and why procedural trade-offs differ from standard aortic valve surgery [22]. Because most comparative datasets are observational and subject to confounding by indication, causal inference remains limited; nevertheless, the recurring direction of evidence across reports aligns with the clinical rationale that reducing direct ascending-aortic manipulation may improve early safety in selected patients [23].

While porcelain aorta primarily raises concerns about ascending aortic manipulation, many affected patients also have systemic atherosclerosis, including peripheral arterial disease (PAD) [24]. Consequently, procedural risk in TAVI often depends on access strategy: transfemoral access is generally preferred when feasible, whereas unfavorable iliofemoral anatomy (often reflecting diffuse PAD) increases the likelihood of vascular complications, bleeding, and unplanned access-site interventions [25]. Several cohorts highlight this “risk trade-off,” whereby the neurologic and aortic-manipulation advantages of TAVI may be partially offset when alternative (non-transfemoral) access is required—particularly in patients with extensive vascular disease [26].

Contemporary TAVI practice has evolved to mitigate access-related complications through meticulous CT-based access planning, smaller delivery profiles, improved closure techniques, and broader use of alternative access routes. When iliofemoral anatomy is unsuitable, transaxillary/subclavian and transcarotid approaches are increasingly used to preserve a transcatheter strategy while avoiding heavily diseased peripheral segments. In porcelain aorta, this access planning is particularly important because it determines whether a patient can realize the potential benefit of an aortic “no-touch” strategy without incurring excessive vascular risk elsewhere [27].

A central challenge in younger and/or low-risk patients with porcelain aorta is balancing short-term procedural safety with lifetime valve management. Because porcelain-aorta-specific long-term follow-up is limited and often fragmented, durability counseling frequently relies on benchmarks from randomized trials and extended follow-up studies in low-risk populations that are not porcelain-aorta-exclusive. Within these benchmarks, long-term follow-up from NOTION and five-year follow-up from PARTNER 3 provide generally reassuring durability and performance signals for contemporary transcatheter valves in selected low-risk patients. Although these trial-derived data cannot be directly extrapolated to porcelain-aorta cohorts without caution, they offer an evidence-based reference point when counseling younger patients in whom future coronary access, valve-in-valve feasibility, and the likelihood of reintervention are clinically relevant. In practice, the porcelain-aorta phenotype may influence the preferred initial strategy (often favoring TAVI to minimize aortic manipulation), while lifetime planning shapes how TAVI is performed (e.g., valve selection and deployment strategy) and how follow-up is structured [28,29].

Hemodynamic performance after valve implantation is particularly relevant in anatomically challenging settings. Across reports with echocardiographic follow-up, TAVI commonly yields favorable gradients and effective orifice areas, a profile that may reduce prosthesis–patient mismatch—especially in smaller annuli or rigid roots that can coexist with extensive calcification. However, hemodynamic comparisons across TAVI and surgical series require caution because valve types, sizing approaches, follow-up timing, and reporting standards vary substantially; thus, these findings should be considered supportive rather than definitive for individualized decision-making [29].

Cerebral embolic protection has attracted attention in porcelain aorta because the embolic substrate may be enriched and embolic vulnerability is a persistent mechanistic concern [30]. Evidence is best interpreted by separating clinical neurological endpoints (disabling/non-disabling stroke) from surrogate endpoints (e.g., new diffusion-weighted MRI lesions or transcranial Doppler signals), which are variably defined across studies [31]. In broader TAVI populations, randomized data show that embolic protection has more consistent effects on surrogate measures than on overall clinical stroke, and limited power and low absolute event rates complicate inference for hard neurological outcomes. Accordingly, in porcelain aorta, embolic protection can be considered to be one element of a broader embolic-mitigation strategy, particularly when CT demonstrates extensive calcific burden or when procedural complexity is expected to increase embolic potential.

Taken together, the literature supports a coherent clinical narrative: porcelain aorta is an anatomic risk modifier in which procedural risk is closely linked to the overall burden of ascending-aortic and great-vessel manipulation. TAVI offers an intuitive advantage by avoiding cross-clamping and direct cannulation of the ascending aorta and is therefore frequently favored when anatomy permits a safe transcatheter access strategy. At the same time, the evidence base remains heterogeneous and largely non-randomized, so patient-centered decisions should be anchored in Heart Team evaluation incorporating CT-defined calcification patterns, access feasibility, and lifetime management principles—particularly in younger and/or lower-risk patients in whom future coronary access and reintervention planning are central.

## 4. Discussion

Even when patients with porcelain aorta (PA) lack severe systemic comorbidities, the technical constraints imposed by a heavily calcified ascending aorta fundamentally change procedural risk. Standard surgical risk models primarily capture age, renal function, ventricular performance, and overall comorbidity burden, and therefore may under-represent localized anatomic hazards such as extensive ascending aortic calcification [4]. In this setting, routine components of surgical aortic valve replacement (SAVR)—including aortic cannulation, cross-clamping, and aortic manipulation—can become disproportionately high-risk, with clinically important threats of embolic stroke, aortic injury, and major bleeding [22].

To mitigate these hazards, surgeons have historically employed modified strategies such as “no-touch” approaches and deep hypothermic circulatory arrest (DHCA) [14]. Although such techniques can reduce the need for direct clamping, they remain invasive and physiologically demanding, and they do not abolish embolic risk. Even when cross-clamping is avoided, embolic sources can persist through arterial cannulation and cardiopulmonary bypass (CPB)-related turbulence and shear forces within rigid, heavily calcified great vessels. DHCA may additionally introduce downstream risks related to prolonged operative complexity and hemostatic disturbance, reinforcing that surgical risk in PA is shaped by more than the simple “clamp versus no clamp” distinction [32].

In contrast, transcatheter aortic valve implantation (TAVI) alters the procedural risk profile by avoiding direct ascending-aortic cannulation and cross-clamping. In PA-specific observational cohorts, neurological event rates after TAVI are frequently reported as low, and several reports describe a directional signal favoring TAVI over surgical strategies that require substantial ascending-aortic manipulation. However, these comparisons remain limited by confounding by indication and heterogeneity in neurological endpoint definitions and adjudication. Accordingly, the most defensible interpretation is not that TAVI “proves superiority,” but that the accumulated observational evidence repeatedly aligns with a biologically plausible principle: reducing direct manipulation of a heavily calcified ascending aorta may reduce embolic hazard [3,13].

Concerns about long-term transcatheter valve durability have historically constrained TAVI use in younger patients and those with longer life expectancy, including patients with PA in whom calcification can be extensive. Yet longer-term benchmarks in low-risk populations increasingly provide reassurance. Long-term follow-up from NOTION and extended follow-up from PARTNER 3 support generally favorable durability and performance signals for contemporary transcatheter valves in selected low-risk patients. While these trial-derived data are not PA-exclusive and should be extrapolated cautiously, they remain the most robust durability reference points when counseling younger patients in whom reintervention risk, valve-in-valve feasibility, and long-term planning are central [28,29].

Cerebral embolic protection (CEP) has also gained attention as an adjunct in embolically vulnerable phenotypes. Randomized evidence in broader TAVI populations suggests CEP can reduce disabling stroke, although effects on overall stroke and surrogate imaging endpoints are less uniform across studies and designs. In practice, CEP can be considered in PA as part of a broader embolic-mitigation strategy—particularly when CT demonstrates extensive calcific burden or when procedural steps are anticipated to increase embolic potential [30].

Access strategy is another determinant of overall risk, because PA often coexists with diffuse atherosclerosis and peripheral arterial disease, increasing the likelihood of iliofemoral limitations. Contemporary guidance supports transaxillary/subclavian and transcarotid approaches when transfemoral access is unsuitable, enabling a transcatheter strategy while avoiding heavily diseased peripheral segments [24]. Ultimately, decision-making in PA should be anatomy-informed and Heart Team-driven, integrating CT-defined calcification patterns, access feasibility, embolic-risk mitigation, and lifetime management principles—particularly in younger or lower-risk patients in whom future coronary access and reinterventions are highly consequential [12].

## 5. Limitations

This state-of-the-art narrative review has several limitations. First, most of the included studies were observational or registry-based, introducing potential selection bias and limiting causal inference. Second, although all cohorts relied on computed tomography imaging, definitions of porcelain aorta varied across studies with respect to extent and severity of calcification. Third, heterogeneity in study design, comparator strategies, outcome definitions, and follow-up duration precluded quantitative meta-analysis. Long-term valve durability data were primarily extrapolated from randomized trials in low-risk populations that did not specifically target patients with PA. While these data represent the most robust available benchmarks for contemporary transcatheter platforms, their applicability to anatomically hostile aortic phenotypes must be interpreted cautiously. In addition, reporting of adjunctive strategies such as cerebral embolic protection and commissural alignment was inconsistent across studies. Nevertheless, the consistency of findings across cohorts and mechanistic investigations supports the validity of the overarching conclusions.

## 6. Conclusions

Porcelain aorta represents an anatomy-driven high-risk phenotype that is not adequately captured by conventional surgical risk scores and that substantially amplifies the neurological and technical hazards of surgical aortic manipulation. Across contemporary studies, TAVI in patients with MSCT-confirmed porcelain aorta is often associated with favorable early outcomes, particularly with respect to stroke and peri-procedural safety, compared with surgical strategies requiring cannulation and/or manipulation of a severely calcified ascending aorta. Although long-term durability data specific to porcelain aorta remain limited, available low-risk trial benchmarks and long-term observational evidence do not indicate an increased risk of structural valve deterioration with contemporary transcatheter platforms. In younger and low-risk patients with porcelain aorta, clinical decision-making should therefore prioritize anatomy-based procedural safety while embedding the index intervention within a comprehensive lifetime management strategy. Procedural planning should emphasize preservation of future coronary access, appropriate access selection in the presence of peripheral arterial disease, and consideration of cerebral embolic protection given the high burden of calcific embolic debris in this population. In this context, TAVI should be regarded not as an alternative to surgery, but as a strategy often favored when ascending aortic manipulation is unsafe.

## Data Availability

The original contributions presented in this study are included in the article. Further inquiries can be directed to the corresponding author.

## Appendix A. Search Strategies (Reproducible Syntax)

PubMed (MEDLINE) (last run 31 January 2026): ((“porcelain aorta”[Title/Abstract] OR “hostile aorta”[Title/Abstract] OR “ascending aorta calcification”[Title/Abstract] OR (“aortic”[Title/Abstract] AND calcification[Title/Abstract])) AND (“transcatheter aortic valve”[Title/Abstract] OR TAVI[Title/Abstract] OR TAVR[Title/Abstract]) AND (low-risk[Title/Abstract] OR “low risk”[Title/Abstract] OR young[Title/Abstract] OR younger[Title/Abstract] OR non-elderly[Title/Abstract])) Filters: Humans; English; Publication date from 1 January 2015 to 31 January 2026. EMBASE (last run 31 January 2026; via Elsevier): (‘porcelain aorta’/exp OR ‘hostile aorta’:ti,ab OR ‘ascending aorta calcification’:ti,ab) AND (‘transcatheter aortic valve implantation’/exp OR tavi:ti,ab OR tavr:ti,ab) AND (‘low risk’:ti,ab OR young*:ti,ab OR ‘non elderly’:ti,ab) AND [humans]/lim AND [english]/lim AND [2015–2026]/py CENTRAL (Cochrane Library) (last run 31 Jan 2026): (porcelain NEXT aorta) OR (“hostile aorta”) OR (“ascending aorta” NEXT calcification) in Title Abstract Keyword AND (TAVI OR TAVR OR “transcatheter aortic valve”) in Title Abstract Keyword; limited to 2015–2026; humans; English.
